# Model-driven discovery of synergistic inhibitors against *E. coli* and *S. enterica* serovar Typhimurium targeting a novel synthetic lethal pair, *aldA* and *prpC*

**DOI:** 10.3389/fmicb.2015.00958

**Published:** 2015-09-23

**Authors:** Ramy K. Aziz, Valerie L. Khaw, Jonathan M. Monk, Elizabeth Brunk, Robert Lewis, Suh I. Loh, Arti Mishra, Amrita A. Nagle, Chitkala Satyanarayana, Saravanakumar Dhakshinamoorthy, Michele Luche, Douglas B. Kitchen, Kathleen A. Andrews, Bernhard Ø. Palsson, Pep Charusanti

**Affiliations:** ^1^Department of Microbiology and Immunology, Faculty of Pharmacy, Cairo UniversityCairo, Egypt; ^2^Department of Bioengineering, University of California, San DiegoLa Jolla, CA, USA; ^3^Computer-Aided Drug Discovery, Albany Molecular Research, Inc., AlbanyNY, USA; ^4^Biology and Pharmacology, Albany Molecular Research Singapore Research Centre, Pte. Ltd., SingaporeSingapore; ^5^The Novo Nordisk Foundation Center for Biosustainability, Technical University of DenmarkHørsholm, Denmark

**Keywords:** synthetic lethality, antibiotic development, drug discovery, systems biology, metabolic reconstruction, bacterial metabolism, model-based drug target discovery, pathway gap filling

## Abstract

Mathematical models of biochemical networks form a cornerstone of bacterial systems biology. Inconsistencies between simulation output and experimental data point to gaps in knowledge about the fundamental biology of the organism. One such inconsistency centers on the gene *aldA* in *Escherichia coli*: it is essential in a computational model of *E. coli* metabolism, but experimentally it is not. Here, we reconcile this disparity by providing evidence that *aldA* and *prpC* form a synthetic lethal pair, as the double knockout could only be created through complementation with a plasmid-borne copy of *aldA*. Moreover, virtual and biological screening against the two proteins led to a set of compounds that inhibited the growth of *E. coli* and *Salmonella enterica* serovar Typhimurium synergistically at 100–200 μM individual concentrations. These results highlight the power of metabolic models to drive basic biological discovery and their potential use to discover new combination antibiotics.

## Introduction

Metabolic network reconstructions are systems biology tools that capture in one framework the function of all known genes, proteins, and reactions within the metabolic network of an organism (Palsson, 2011). The conversion of a metabolic reconstruction into a computational format allows one to simulate flux states of the network that correspond biologically to different phenotypes, and thereby to computationally investigate the genotype–phenotype relationship for an organism. The ability to simulate different phenotypes distinguishes computational models from static maps of biochemical pathways. The latter provides a pictorial diagram of all pathways in a network but no information on their usage or activity levels in a living organism, while the former provides information regarding which pathways are actually active under the simulation condition. Different sets of pathways will be active under different conditions, a situation referred to as the functional states of the network.

One application of metabolic reconstructions and their associated mathematical models is to drive new biological discovery (Chen and Vitkup, 2006; Reed et al., 2006; Orth and Palsson, 2012). The workflow consists of a loop in which simulation output is first compared to experimental data to find inconsistencies between the two. Hypotheses are then formed to reconcile the two data types and subsequently tested in the lab. New information gleaned from the experiments is then incorporated into the model, after which a new cycle of simulation, testing, and model refinement begins. Each cycle improves the accuracy, predictive ability, and therefore utility of metabolic models. Metabolic models have already been used to aid strain design for metabolic engineering (Lee et al., 2005; Perez Pulido et al., 2005; Park et al., 2011; Licona-Cassani et al., 2012), to analyze network properties (Almaas et al., 2005; Nam et al., 2012), and to provide context for the analysis of high-throughput omics data (Chandrasekaran and Price, 2010; Chang et al., 2010; van Berlo et al., 2011), and their role in such projects is anticipated to grow as the models are refined and simulate a larger number of biological conditions more accurately.

As another application, we recently used metabolic models of four species of Enterobacteriaceae to guide the search for synthetic lethal (SL) gene pairs in these bacteria (Aziz et al., Submitted). Metabolic models accelerate this search since they can compute the impact of all double deletion mutants on growth. Gene pairs that result in *in silico* synthetic lethality become candidates for experimental validation. In this way, metabolic models narrow a very large search space of all possible double deletion mutants down to a more focused subset. For *Saccharomyces cerevisiae*, the use of a metabolic model improved the search for SL gene pairs by two orders of magnitude over random selection of gene pairs (Harrison et al., 2007). Validated SL pairs are significant because they are potential drug targets, for example as targets for antibiotic development against pathogenic bacteria, since the inhibition of both enzymes leads to cell death. Metabolic models consequently form a potential bridge from basic biology to translational medicine by facilitating the discovery of a specific class of combination therapeutics. Drug combinations have garnered interest in antibacterial drug discovery since recent studies suggest that the number of single drug targets in bacteria is limited (Becker et al., 2006; Payne et al., 2007).

In the current version of the *Escherichia coli* metabolic reconstruction (Orth et al., 2011), there is an inconsistency that centers on the gene *aldA*: it is essential for *in silico* growth in glucose M9 medium, but the knockout mutant is viable experimentally. Here, we present experimental data suggesting that *aldA* is not singly essential because it forms an SL pair with *prpC*. We also perform virtual and biological screening against these two enzymes to search for sets of compounds that inhibit growth of the bacteria, and find one set that inhibits the growth of *E. coli* and *Salmonella enterica* serovar Typhimurium (hereafter referred to *S.* Typhimurium) synergistically at 100–200 μM concentrations.

## Materials and Methods

### Bacterial Strains and Media

*Escherichia coli* wild-type, *E. coli* Δ*aldA*, and *E. coli* Δ*prpC* were taken from the Keio collection (Baba et al., 2006) and used for construction of the Δ*aldA* Δ*prpC* mutant via complementation. An *E. coli* K12 MG1655 Δ*aldA* was used for transposon mutagenesis. A third strain of *E. coli* was used for biological screening and is noted below. All strains were grown in either Luria-Bertani (LB) broth/agar or glucose M9 media. The M9 medium contained 2 g/L glucose, 100 μM CaCl_2_, 2 mM MgSO_4_, 6.8 g/L Na_2_HPO_4_, 3 g/L KH_2_PO_4_, 0.5 g/L NaCl, 1 g/L NH_4_Cl, and 250 μL/L trace elements. The trace element solution consisted of (per liter): FeCl_3_⋅6H_2_O (16.67 g), ZnSO_4_⋅7H_2_O (0.18 g), CuCl_2_⋅2H_2_O (0.12 g), MnSO_4_⋅H_2_O (0.12 g), CoCl_2_⋅6H_2_O (0.18 g), and Na_2_EDTA⋅2H_2_O (22.25 g). Antibiotics were added as necessary at the following concentrations: ampicillin at 100 μg/mL, kanamycin at 50 μg/mL, and chloramphenicol at 25 μg/mL. LB powder was purchased from EMD Chemicals (Gibbstown, NJ, USA) and used at the manufacturer’s recommended concentration. All other chemicals were purchased from Fisher Scientific (Waltham, MA, USA) or Sigma-Aldrich (St. Louis, MO, USA).

### Growth Rate Determination

The Bioscreen C instrument (Oy Growth Curves Ab Ltd., Finland) was used to measure the optical density of the samples for growth rate calculations. Briefly, 400 μL samples of each strain were pipetted into separate wells in triplicate. The initial OD600 was 0.05. The plate was then placed into the instrument, and OD600 measurements taken every 15 min over 48 h. The plate was shaken for 10 s prior to each reading, and the incubation temperature was 37°C. The growth rate was calculated in Microsoft Excel as the slope of the straight line that was best fit to the logarithm of the OD600 values during the exponential growth phase.

### Metabolic Modeling and Prediction of Single Gene Essentiality

Metabolic network reconstructions for *E. coli* K12 MG1655 (Orth et al., 2011) were loaded into the COBRA Toolbox (Ebrahim et al., 2013). Default bounds were retained for all model reactions (Orth et al., 2011). To simulate glucose M9 and LB media conditions, we adjusted the lower bound of each exchange reaction according to the media composition (Aziz et al., Submitted). For example, lower bounds for M9 minimal media were set at -1000 (allowing unlimited uptake) on the exchange reactions for Ca^2+^, Cl^-^, CO_2_, Co^2+^, Cu^2+^, Fe^2+^, Fe^3+^, H^+^, H_2_O, K^+^, Mg^2+^, Mn^2+^, MoO_4_^2-^, Na^+^, Ni^2+^, SeO_4_^2-^, SeO_3_^2-^, and Zn^2+^ as previously detailed (Monk et al., 2013). Single knockout mutants were modeled by using the delete_model_gene function to constrain each reaction catalyzed by the corresponding enzyme to zero. Model growth phenotypes were determined using flux balance analysis (FBA) with the core biomass reaction as the objective. If a particular knockout resulted in a simulated growth rate equal to zero, that gene was deemed to be singly essential. The Gurobi (Gurobi Optimizer Version 5.6, Gurobi Optimization, Inc.) linear programming solver was used to perform FBA.

### Transposon Mutagenesis

To create the Δ*aldA* transposon library, we grew a freshly inoculated colony of the Δ*aldA* knockout strain to an optical density of 0.8 in LB at 37°C, then harvested the cells and made them competent by washing three times in chilled 10% glycerol. Fifty microliters of the washed cell pellet was transformed with 1 μL of EZ-Tn5 transposome (Epicenter, Illumina, USA) by electroporation (BioRad electroporator at 2500 V), and the electroporated cells were recovered by incubation in SOC media to a final volume of 1 mL. An aliquot of the library was tested for the presence of transposon and for purity. Tn5-transformed cells were selected as single colonies on LB agar containing 50 μg/mL kanamycin; no heterogeneous colonies were observed.

The genetic loci that were disrupted by the EZ-Tn5 transposon were identified by the rapid amplification of transposon ends (RATEs) method (Ribot et al., 1998). Briefly, DNA from selected colonies was amplified by a three-stage PCR: the first stage is a unidirectional primer extension reaction using inverse primers that linearly amplify segments of random lengths corresponding to the inserted transposon ends. The second stage is a permissive PCR that uses the same primers non-specifically to generate random double stranded fragments of the two transposon ends extended in the first stage. The third stage is now a highly specific PCR that amplifies the random-length double-stranded fragments, which include portions of the two insertion sites, which are of enough length to determine the identity of those insertion sites by Sanger sequencing.

### Construction of Knockout Mutants

All gene knockouts were created using the protocol of Datsenko and Wanner (2000). Briefly, a kanamycin resistance cassette containing flanking FRT sites was generated by PCR using pKD13 as the template. The ends of the cassette comprised 60 nucleotides that contained the start or stop codon plus 57 bp that were homologous to the 57 bp immediately upstream and downstream of gene to be deleted. PCR and Sanger sequencing confirmed correct insertion of the marker and subsequent removal from the chromosome. All PCR products were purified with the QIAGEN PCR clean-up kit (Valencia, CA, USA).

### *aldA* Complementation

The pASK1988 plasmid (Fong et al., 2013), based on pASK-iBA33+ (IBA GmbH, Goettingen, Germany), was used for complementation. The *aldA* gene was inserted into pASK1988 by PCR amplifying the backbone of the plasmid and the *aldA* gene such that the two fragments had 21 or 22 bp overlapping sequences on each end of the two fragments. The two fragments were then mixed together, and PCR cycling used to anneal them together. The plasmid was gel purified, and correct insertion of *aldA* verified by Sanger sequencing. This plasmid was next transformed into the *E. coli* Δ*prpC* via electroporation.

To construct the Δ*prpC* Δ*aldA* mutant, we inoculated 1 mL of an overnight culture of *E. coli* Δ*prpC* bearing the complementation plasmid into each of four Erlenmeyer flasks containing 100 mL LB plus chloramphenicol. Expression of the target gene on this plasmid is normally induced by anhydrotetracycline (aTc). As such, aTc was added at 0, 50, 100, and 200 μg/mL concentrations to the four cultures, and all four were incubated at 30°C for 3–4 h. The four cultures were then washed three times with ice-cold 10% glycerol, after which they were transformed with the *aldA* knockout cassette. Colonies bearing deletions in both *prpC* and *aldA* were confirmed by PCR and Sanger sequencing. Interestingly, the culture in which no aTc had been added was the only one that yielded colonies after transformation, suggesting constitutive rather than inducible expression. The selection marker for the knockout cassette (kanamycin resistance) was then cured from the Δ*prpC* Δ*aldA* mutant using pCP20 following standard protocol (Datsenko and Wanner, 2000). The mutant also regained sensitivity to chloramphenicol, the selection marker for pASK1988; however, a plasmid-borne copy of *aldA* could still be detected by PCR using primers that amplified a region wholly within the chloramphenicol resistance gene.

### Protein Structural Modeling

The only available crystallographic structures for PrpC come from organisms other than *E. coli* (e.g., *Salmonella* Typhimurium). Therefore, a homology model for PrpC was taken from a previously constructed template-based homology model (PRPC_ECOLI) using the I-TASSER suite of programs (Xu and Zhang, 2012, 2013). The structural assessment for AldA is based on a native PDB template, 2hg2 (chain A; Di Costanzo et al., 2007).

### Virtual Screening and Compound Selection

Crystal structures of six proteins were selected from the protein databank (www.rcsb.org), which were identical in sequence to the proteins found in the two bacteria, and in total, 13 sites were used for docking. Preference was given to those structures that contained a co-crystalized substrate or inhibitor so that we could select compounds that likely bind at a catalytic site. Standardized methods were used to prepare each binding site for docking, and to model the sites with compounds that confer appropriate shape and electrostatic interactions for potential inhibitory compounds. Diverse compounds from a library of 300,000 to 600,000 commercial compounds were docked using high throughput virtual screening (HTVS) precision. The best scoring compounds were subjected to atom pair similarity calculations (Carhart et al., 1985) to determine chemically similar structures with a similarity cutoff of 70–80%. In addition, compounds that contained the maximum HierS scaffolds (Wilkens et al., 2005) of any compound on the HTVS list were also selected. The resulting combined list of compounds was docked again using standard precision. Compounds that docked well-according to the Glide docking program and scored well-based on a complementary scoring scheme were examined manually and purchased, if available.

Our selection choices were guided by the need for at least one or two single-agent inhibitors per protein target. Therefore, we set a goal of testing a minimum of 200 compounds per protein target based on reported success rates of selecting true active compounds in typical *in vitro* biochemical screens by docking methods. Docking methods provide a mean enrichment factor of 1–60 over random selections with an enrichment of 10 being a reasonable expectation, or approximately a 10% hit-rate (McGaughey et al., 2007). We also assumed that only ∼10% of compounds would permeate the Gram-negative cell walls yielding a net 1% hit rate in bacterial assays.

After identifying weak hits in the bacterial growth inhibition assay, we selected chemically similar compounds and likewise docked them to their putative binding site. The selection of similar compounds was done using atom pair similarity (Carhart et al., 1985) calculations with a similarity cutoff of 70–80%. These similar compounds were then docked using the same methods as the original compounds. Any resulting compounds that met the same docking score criteria as the original compounds were purchased, if available.

### Primary Compound Screening

The IC_50_ and percentage inhibition of each purchased compound was tested in bacterial growth inhibition assay. *E. coli* (ATCC 25922) and *S. enterica* (ATCC 14028), both purchased from American Type Culture Collection (Manassas, VA, USA), were used for the growth inhibition assays. The strains were propagated on BBL^TM^ Mueller-Hinton II broth (Cation-Adjusted) and BBL^TM^ Mueller-Hinton II agar (Becton, Dickinson and Company, USA). The glycerol stocks were made and stored at -80°C. Colony-forming units (CFUs) were determined for stocks and the assay inoculum to assess initial viability and to ensure consistent assay performance. The bacterial growth inhibition was measured using broth microdilution method. All test compounds were dissolved in DMSO (20 mM) and plated onto 384-well microtitre source plates (Corning, NY, USA) using Biomek FX (Beckman Coulter, USA) in a twofold eight-point dose response series (20 –0.156 mM). The assay plates were stamped from source plates using Cartesian Hummingbird (Digilab, Inc., USA) and each well received 500 nl of the compound. Standard bacterial inoculum of 5 × 10^5^ CFU/mL was used for the assay and 50 μl per well was dispensed using Multidrop (Thermo Fisher Scientific, Inc., USA). The assay plates were incubated at 37°C for 18 h and bacterial growth was measured by absorbance at 600 nm. The IC_50_ of the test compounds were calculated using IDBS XLfit data analysis software. To rule out any assay interference from the test compounds, turbidity assessment for all the compounds in Mueller-Hinton II broth was conducted at 600 nm. None of the compounds exhibited significant absorbance at 600 nm. The assay performance was monitored for each assay plate and Z’ scores of ≥0.5 and coefficient of variation (CV) of ≤10% for positive and negative growth controls included in each assay plate were kept as assay acceptance criteria. In addition, reference antibiotics including amikacin, azithromycin, ceftriaxone, chloramphenicol, levofloxacin, and tetracycline (Sigma-Aldrich, USA) were tested on the growth inhibition assay as controls on each assay day. The minimum inhibitory concentrations (MICs) were determined for these reference antibiotics using standard Clinical and Laboratory Standards Institute (CLSI) method. The assay plates were failed and retested if they did not meet the assay acceptance criteria or if the shift in the obtained MIC values of the reference antibiotics for any strain/antibiotic combination exceeded >2-fold.

### Combination Studies

A preliminary combination study was performed using four compounds (ALDA-112, ALD-087, ALDA-70, and PRPC-034) selected from the primary screening. Each compound was tested in combination at two by two concentrations. Two concentrations of each compound that caused 25 and 50% growth inhibition of *E. coli* and/or *S.* Typhimurium were chosen for the combination study. Based on the results of the primary combination studies, three compounds were further shortlisted for combination studies using eight by eight test concentrations (top test concentration of 200 μM). The bacterial growth inhibition assay was performed by methods as described in the previous section. All the experiments were performed in duplicates.

## Results

### In Contrast to Simulation Data, an *E. coli ΔaldA* Mutant is Viable

**FIGURE 1:**
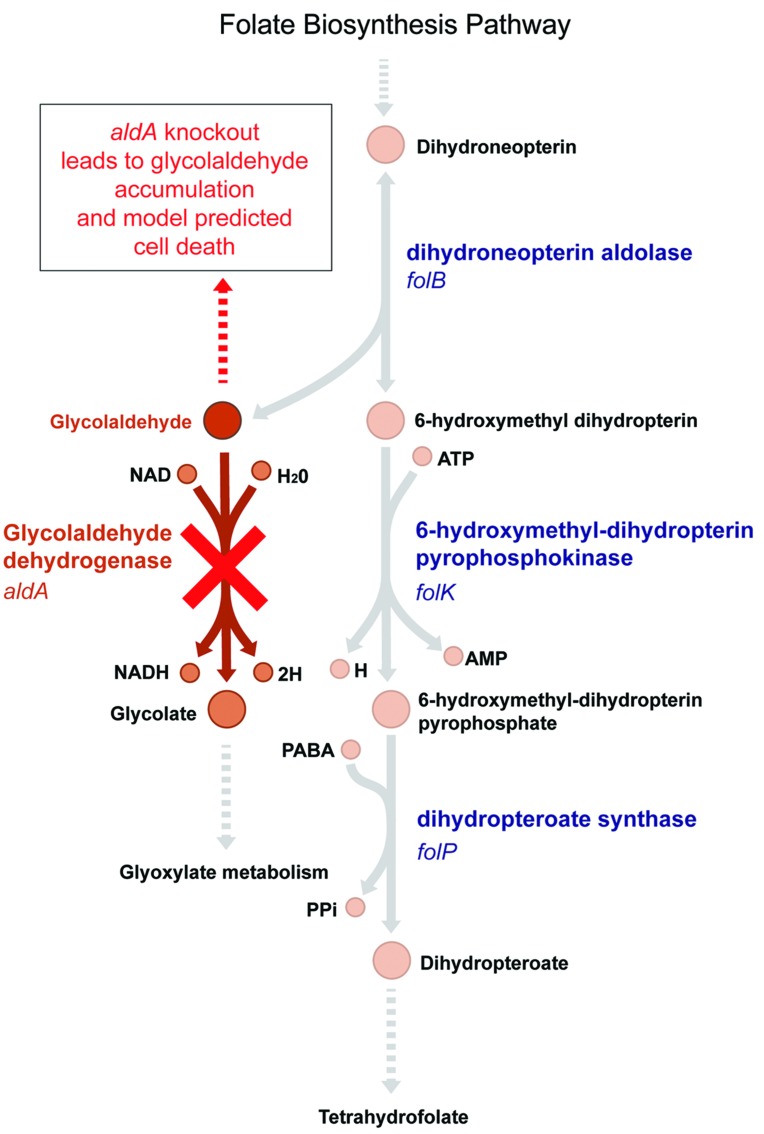
**The model-predicted essential pathway catalyzed by AldA.** Glycolaldehyde is a by-product of the essential tetrahydrofolate synthesis pathway in *Escherichia coli*. It is produced by dihydroneopterin aldolase, encoded by the gene *folB*. The *aldA* gene encodes glycolaldehyde dehydrogenase, which oxidizes glycolaldehyde to glycolate. In an Δ*aldA* knockout strain, the *E. coli* metabolic model predicts that glycolaldehyde will accumulate in the cell, leading to cell death (Orth et al., 2011). Experimentally, however, Δ*aldA* mutants are viable.

Simulations using the most recent version of the *E. coli* metabolic model (Orth et al., 2011) suggest that *aldA* should be an essential gene in glucose M9 media; however, the Δ*aldA* mutant is viable experimentally in this medium (Supplementary Figure S1). Glycolaldehyde dehydrogenase A (AldA), encoded by *aldA*, is an enzyme of broad specificity for small α-hydroxyaldehyde substrates (Baldoma and Aguilar, 1987). It is known to oxidize L-lactaldehyde to L-lactate in the metabolic pathways for L-fucose and L-rhamnose utilization, and catalyzes glycolaldehyde dehydrogenation of different pentoses such as D-arabinose and L-lyxose (LeBlanc and Mortlock, 1971; Badia et al., 1991). This latter function (and its encoding gene *aldA*) is predicted by the model to be essential for growth in glucose M9 because of its role in the folate biosynthesis pathway. In order for the model to synthesize folate, an essential metabolite, dihydroneopterin aldolase (FolB) must convert dihydroneopterin to 6-hydroxymethyl dihydropterin, which produces glycolaldehyde as a by-product. AldA then converts glycolaldehyde to glycolate (**Figure 1**). Without this reaction, an infinite amount of glycolaldehyde would accumulate in the model, which is an infeasible solution because it violates mass balance. Biologically, aldehydes are electrophilic compounds that are often toxic. Therefore accumulation of glycolaldehyde during the synthesis of folate due to an *aldA* gene disruption could be lethal to growth of *E. coli*. The *E. coli* Δ*aldA* mutant is viable in glucose M9, however, suggesting that either glycolaldehyde diffuses out through the membrane or is converted to glycolate by a different enzyme.

### *aldA* and *prpC* Appear to be Synthetically Lethal

Based on the inconsistency between simulation and experimental data, we hypothesized that an unidentified enzyme could catalyze the same essential reaction currently assigned only to AldA. To test this hypothesis, we used transposon mutagenesis to introduce random gene disruptions in the Δ*aldA* background and screened for the absence of non-essential genes in the transposon library. To verify these data, we subsequently attempted to create 15 double deletion mutants, each comprising *aldA* and one of the top 15 hits from the screen. We could successfully create fourteen of these double mutants and grow them in glucose M9 (Supplementary Table S1). Despite repeated attempts, however, we failed to isolate a Δ*aldA*Δ*prpC* double mutant, even in LB medium.

**FIGURE 2:**
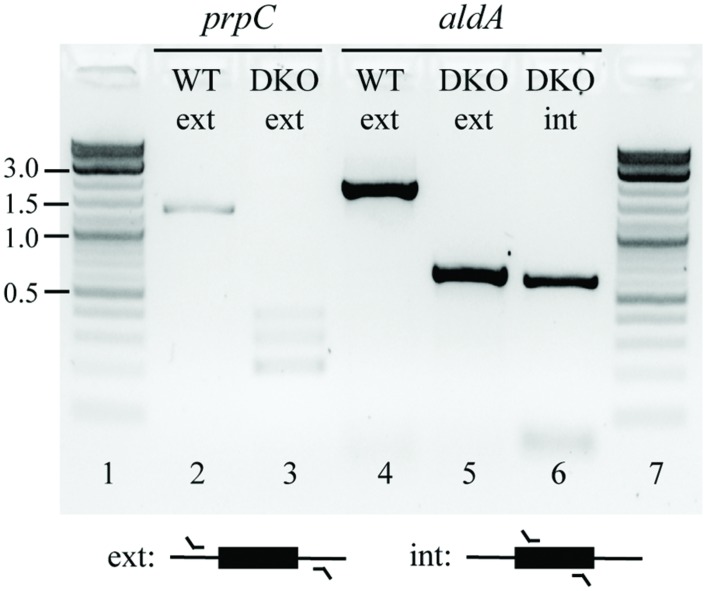
**Confirmation of the Δ*prpC* Δ*aldA* deletions at their annotated chromosomal positions, and the continued presence of a copy of *aldA* at an undefined location.** Colonies were isolated in which the chromosomal copies of *prpC* and *aldA* had been successfully deleted (lanes 3 and 5 versus lanes 2 and 4, respectively), but PCR amplification using primers that targeted a region wholly within *aldA* revealed that a copy of this gene was still present in the double mutant (lane 6). The identity of this amplicon was also confirmed by Sanger sequencing. Abbrevations: WT, wild-type; DKO, Δ*prpC* Δ*aldA* double knockout; ext, external; int, internal. Numbers on the far left indicate DNA band sizes in kb.

Based on this outcome, we hypothesized that *aldA* and *prpC* might form a SL gene pair in both LB and glucose M9. To further investigate this possibility, we cloned *aldA* into the pASK1988 overexpression vector (Fong et al., 2013), transformed it into a Δ*prpC* mutant, and re-attempted to create the double knockout. With this complementation, we could successfully delete the chromosomal copy of *aldA*. We next removed the kanamycin selection marker and attempted to cure the overexpression plasmid from the double mutant. All colonies regained sensitivity to the selection marker present on the complementation plasmid (chloramphenicol resistance), but the presence of *aldA* could still be detected within the double mutant (**Figure 2** and Supplementary Figure S2). These data support the hypothesis that *aldA* and *prpC* are synthetically lethal.

### Structural Comparison between AldA and PrpC

**FIGURE 3:**
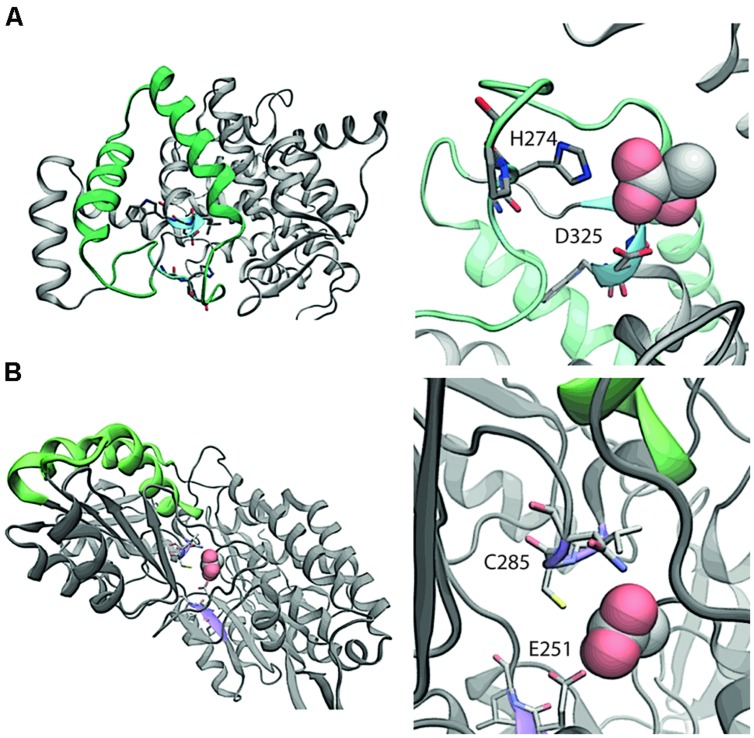
**Comparative structural analysis of (A) PrpC and (B) AldA.** In the left panel of **(A)**, the region with the highest overlapping structural similarity between PrpC and AldA is shown in green. The putative binding site with catalytic residues H274 and D325 are shown in blue. In the native reaction, 2-methyl citrate synthase, oxaloacetate reacts with a CoA-ester (e.g., propionyl-CoA) to form (2R,3S)-2-hydroxybutane-1,2,3-tricarboxylate. The NADH binding site is 15 Å away from the closest residue in the green overlapping region, and is therefore not considered a part of the overlap. Shown in the right panel of **(A)** is a magnification of the putative binding site. In the left panel of **(B)**, the region with the highest overlapping structural similarity is again shown in green. The putative binding site (violet) shows where lactic acid binds to the protein. The putative binding orientation of lactic acid is taken from an alignment with PDB entry 3o8j.

We compared the protein structures for AldA and PrpC to investigate whether PrpC might have unrecognized promiscuous dehydrogenase activity. AldA is comprised of 61.3% alpha helix/beta sheet content versus 58.8% for PrpC, and the two differ in length by 90 amino acids (479 versus 389 for AldA and PrpC, respectively, **Figure 3**). A pairwise comparison using DALI (Holm and Rosenstrom, 2010), however, indicates very little sequence and secondary structural similarity. Only 47 of the 479 (10%) residues in AldA have structural similarity to residues in PrpC, and PrpC lacks any indication of an NADH binding site (**Figure 3**). The likelihood that PrpC exhibits promiscuous dehydrogenase activity is therefore low.

### Virtual and Initial Biological Screening of Drug-Like Molecules against AldA and PrpC Identified Three Compounds with Potential to Elicit Combination Effects

The protein products of validated SL pairs are potential drug targets, and small molecule inhibitors against them are expected to inhibit growth synergistically when the two compounds are present in combination but have little to no inhibitory effect when they are present individually. Synergy is an attractive feature in drug combinations (Lehar et al., 2009). We therefore evaluated the feasibility of finding synergistic inhibitors based on synthetic lethality by performing virtual and biological screening against AldA and PrpC. Compounds were initially screened to identify those that inhibit bacterial growth weakly as single agents, after which they were tested in combination with each other to assess potential synergy, additivity, and antagonism among the molecules.

**FIGURE 4:**
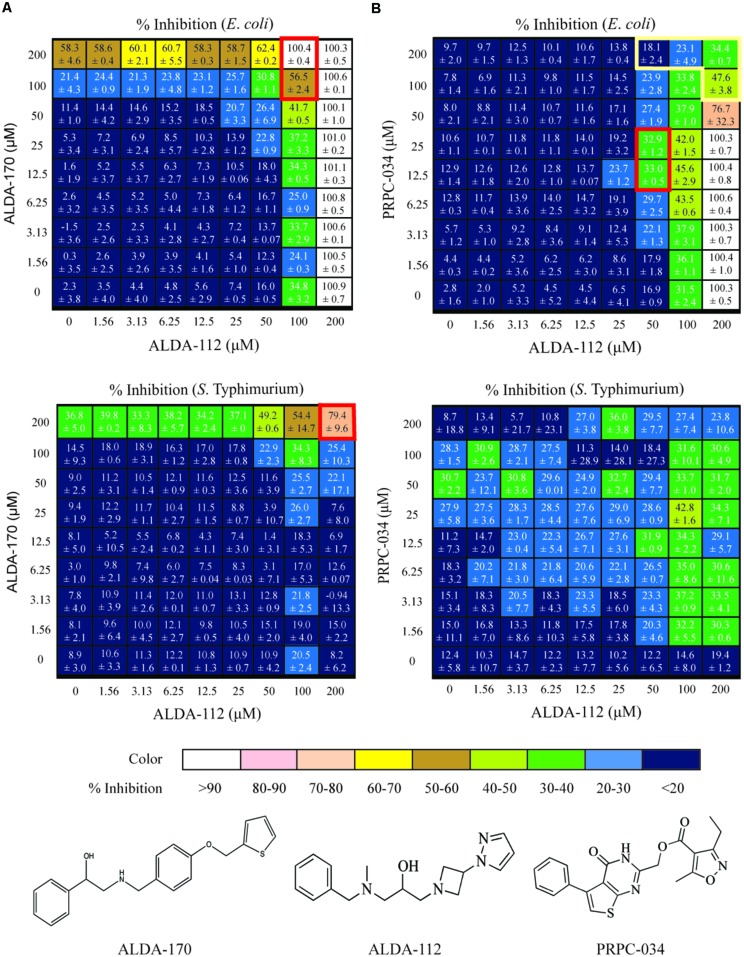
**Dose response matrix for ALDA-112/ALDA-170, and ALDA-112/PRPC-034 against *E. coli* and *S.* Typhimurium.** The single agent effects are depicted in the far left column and along the bottom row of each matrix. Each square represents a unique combination of the indicated compounds, and the number represents the level of growth inhibition that was measured plus standard deviation. **(A)** The ALDA-112/ALDA-170 pair exhibits mostly additivite inhibition against both *E. coli* and *S.* Typhimurium, with synergy at 100–200 μM concentration (red boxes). **(B)** Likewise, ALDA-112/PRPC-034 primarily exhibits additive inhibition at most concentrations against *E. coli* but there is one region of synergy (red box) and another region of antagonism (yellow box). No discernible pattern against *S.* Typhimurium is observed.

Two hundred seventy-three compounds were identified from virtual screening against AldA and PrpC. The selected compounds were tested in an eight-point dose response format (top test concentration of 200 μM and twofold dilution) against *E. coli* and *S.* Typhimurium to determine their IC_50_ and percent inhibition (where IC_50_ could not be established) against the two bacteria. Although the virtual screening was carried out against protein targets, biological screening was performed as a growth inhibition assay in which the readout was bacterial growth. For all bacterial growth assay plates, the Z’ scores were greater than 0.5, and the percent CV for positive and negative controls on each assay plate were less than 10%. Furthermore, the MIC data generated with the reference antibiotics for each strain were consistent between multiple experimental days, confirming uniformity across the screening campaign. Two compounds targeting AldA, ALDA-112 and ALDA-170 exhibited growth inhibition against *E. coli* with an IC_50_ of 108 and 200 μM, respectively (**Table 1**). Two additional compounds, one targeting AldA (ALDA-087) and one targeting PrpC (PRPC-034), showed weaker activity against both *E. coli* and *S.* Typhimurium (>20% inhibition at 200 μM). IC_50_ values could not be determined for these two compounds due to their weak activity. The structures are shown in **Figure 4** and Supplementary Figure S3.

**Table 1:** Results of antimicrobial screening against *E. coli* and *S.* Typhimurium.

		Number of active compounds			
		*Escherichia coli*		*Salmonella* Typhimurium	
Target protein	Number of compounds tested	IC_50_ of 0.1–200 μM	>30% inhibition at 200 μM	IC_50_ of 0.1–200 μM	>30% inhibition at 200 μM
PrpC	99	0	0	0	0
AldA	174	2	2	0	2

*The table shows the number of compounds tested for each of the target proteins and their respective activity profile against the two bacteria using repurchased and reweighed samples.*

These four compounds were selected for combination studies against both *E. coli* and *S.* Typhimurium. Initially, two concentrations of one compound were tested against two concentrations of another compound to produce a 2x2 matrix for each of the six possible combinations (Supplementary Table S2). Potential synergy, additivity, and antagonism were assessed based on the Loewe model (Lehar et al., 2007). Full dose response testing of the individual compounds was also performed in parallel. The combination of ALDA-112 and ALDA-170 showed a 99.7% (+0.09%) actual percent inhibition compared to the 82.8% expected percent inhibition if the effect was additive (*E. coli*; Supplementary Table S2A) and 42.7% (+0.16%) actual percent inhibition compared to the 40% expected percent inhibition (*S.* Typhimurium; Supplementary Table S2B). Moreover, ALDA-112 and PRPC-034 showed an actual percent inhibition of 24.8% (+5.17%) compared to the 18.1% expected additive percent inhibition against *S.* Typhimurium (Supplementary Table S2B). Consequently, we subjected ALDA-112, ALDA-170, and PRPC-034 to a detailed 8x8 combination study to more fully investigate whether these preliminary 2x2 effects are due to synergy or addtivity (**Figure 4**). The anti-microbial activity of the compounds when used individually was also measured in parallel.

### ALDA-112 and ALDA-170 Inhibit the Growth of *E. coli* and *S.* Typhimurium Primarily in an Additive Manner, but Display Synergy at 100–200 μM Concentration

Against *E. coli*, ALDA-112 by itself inhibited all bacterial growth when tested individually at 200 μM (**Figure 4A**). Because *aldA* is not singly essential, this outcome suggested that ALDA-112 targets a different enzyme other than AldA, or it exerts a non-specific growth inhibitory effect at this concentration. ALDA-170 exhibited approximately 60% (58.3 ± 4.6%) growth inhibition when tested alone at 200 μM, and this effect remained constant (62.4 ± 0.2%) even when ALDA-112 was present at concentrations up to 50 μM. However, the addition of 100 μM ALDA-112 resulted in synergistic inhibition (**Figure 4A**). Against *S.* Typhimurium, ALDA-112 did not inhibit bacterial growth at 200 μM like it did against *E. coli* (only 8.2% growth inhibition), but ALDA-170 again displayed nearly constant growth inhibition when present at this concentration. Together, however, the two compounds exhibited 79 ± 9.6% growth inhibition when they were both present at 200 μM (**Figure 4A**). The two molecules therefore primarily exhibit an additive effect against the two bacteria, but exhibit synergistic inhibition at 100–200 μM.

### ALDA-112 and PRPC-034 Have No Discernible Inhibition Pattern against *S*. Typhimurium

Against *E. coli*, the combination of ALDA-112 and PRPC-034 likewise exhibited primarily additivity, but displayed one pocket of synergy and another pocket of antagonism (**Figure 4B**). In contrast to ALDA-112 and ALDA-170, antagonism was the dominant effect when ALDA-112 and PRPC-034 are present at and above 50–100 μM. Against *S.* Typhimurium, the two compounds displayed no discernible pattern because each compound exhibits low growth inhibition with similar values at all tested concentrations and often within the expected experimental uncertainty.

## Discussion

Genome-scale metabolic network models can be used prospectively to guide biological discovery. As new data are acquired and incorporated, the updated model is used to drive a new round of biological discovery and refinement. This cyclical process ultimately yields models that more accurately simulate experimental outcomes. Here, we traverse one loop of this cycle by presenting data in which apparent synthetic lethality between *aldA* and *prpC* explains why *aldA* is not singly essential, which is the computed phenotype using the most current version of the *E. coli* metabolic model (Orth et al., 2011). We also perform virtual and biological screening against AldA and PrpC, identifying a pair of compounds that inhibit *E. coli* and *S.* Typhimurium synergistically when the two compounds are present at 100–200 μM concentration.

Two different mechanisms could explain the synthetic lethality between *aldA* and *prpC*. First, the PrpC protein might directly replace the catalytic function of AldA; however, this scenario is unlikely as AldA and PrpC have low sequence and structural overlap (**Figure 3**). Second, there might be an uncharacterized pathway that consumes glycoaldehyde and involves PrpC. One possibility is a two step pathway in which glycolaldehyde is first converted to glycolyl-CoA by an unknown coenzyme A-dependent aldehyde dehydrogenase, followed by condensation of glycolyl-CoA into products that enter central metabolism (Supplementary Figure S4). The gene encoding the enzyme catalyzing the first step in this hypothetical pathway would also be synthetically lethal with *aldA*, but no such gene was found in the Tn-mutagenesis screen. This outcome implies that the transposon did not sufficiently cover the genome or that more than one enzyme can catalyze this reaction. For the second step, citrate synthase (GltA) in other organisms has been shown to be capable of condensing glycolyl-CoA with oxaloacetate (Vamecq et al., 1990). Because PrpC has demonstrated citrate synthase activity (Patton et al., 1993) and they appear to be isozymes (Guzman et al., 2015), it is possible that PrpC could be required for the essential condensation of glycolyl-CoA formed in *aldA* knockout strains.

The discovery of *aldA* and *prpC* as a SL pair arose because the most current version of the *E. coli* metabolic model (Orth et al., 2011) computes *aldA* as an essential enzyme, but experimentally it is not. In reality such cases are rare, as metabolic models for *E. coli* compute single gene essentiality with accuracy over 90% (Feist et al., 2007; Orth et al., 2011). The accuracy of prediction for SL gene pairs is lower, but it is still better than if pairs are selected by random chance. For *S. cerevisiae*, for example, the accuracy is 49% using the *S. cerevisiae* iLL672 metabolic model, a rate that is two orders of magnitude better than if two genes are picked randomly (Harrison et al., 2007). The rate is likely similar for *E. coli*. In this context, improving the accuracy with which metabolic models compute the phenotypic outcome of gene–gene interactions represents the next stage of refinement for metabolic models. Similar to single gene experiments, the workflow would likely consist of simulating the phenotype for large numbers of double deletion mutants and comparing the results to experimental data. Inconsistencies between the two then become the foundation for further experimental testing and model refinement.

Small molecules that inhibit both members of a SL protein pair are hypothesized to do so synergistically. Synergy among different components in a drug combination is an advantageous property as it can lead to reduced dosages of the individual compounds and be more specific to a particular cellular context (Lehar et al., 2009). Our screening campaign yielded one pair of compounds that displayed primarily additive inhibition when present below 100 μM, while synergy appears above this threshold. These molecules were identified in a virtual screen against the target proteins but the biological assay was based on bacterial growth. Therefore, the observed synergy could be due to other factors. In addition, factors such as cell permeability and eﬄux could decrease the synergistic effect. Additional work is warranted to assess whether the molecules do in fact target AldA and PrpC in a cellular context. These data point to the difficulty of transferring data from a target-centric screen, implemented here through virtual screening, to whole-cell assays. Future studies could be modified to better account for these factors. For example, compounds identified through virtual screening as PrpC inhibitors could be screened against both the wild-type bacterium and a Δ*aldA* mutant. This screen would be repeated for AldA inhibitors and Δ*prpC* mutants. Compounds showing more sensitivity in individual assays toward the two mutants when compared to the same dosage against the wild-type would then be tested for synergy.

## Conclusion

In this work, we use an inconsistency between simulation and experimental data to drive new biological insight, finding that *aldA* and *prpC* form a SL pair, and to investigate whether this finding might translate into a biomedical application. Furthermore, we identify a pair of compounds through virtual and biological screening that inhibit *E. coli* and *S.* Typhimurium synergistically at 100–200 μM. Follow-up work is needed to confirm that inhibiton of AldA and PrpC is indeed the mechanism of action of these molecules, after which structural refinement based on the core molecular scaffolds might lead to a more potent pair of compounds. More generally, continued refinement of metabolic models to boost their ability to predict gene–gene interactions more accurately would improve their utility across different fields.

## Author Contributions

RA, BP, and PC designed the experiments. RA, VK, and KA constructed and tested the knockout mutants. JM implemented and carried out simulations involving the metabolic models. JM and EB performed the structural analysis. DK designed the docking and compound selection protocols. RL performed the docking calculations and protein modeling. DK, ML, AM, CS, and SD designed the antibacterial combination experiments. SL, AM, AN carried out the antibacterial and combination experiments. All authors contributed to data analysis. RA, JM, EB, BP, DK, RL, SL, AM, CS, SD, and PC contributed to manuscript preparation.

## Conflict of Interest Statement

The authors declare that the research was conducted in the absence of any commercial or financial relationships that could be construed as a potential conflict of interest.
