# Supplementary material for: Model-driven discovery of synergistic inhibitors against E. coli and S. enterica serovar Typhimurium targeting a novel synthetic lethal pair, aldA and prpC
Source: Front Microbiol. 2015 Sep 23;6:958. doi: 10.3389/fmicb.2015.00958 (PMC4585216; doi:10.3389/fmicb.2015.00958)
Supplement: Supplementary file 1 [file Data_Sheet_1.PDF]

SUPPLEMENTARY INFORMATION

Model-driven discovery of synergistic inhibitors against *E. coli* and *S. enterica* serovar Typhimurium targeting a novel synthetic lethal pair, *aldA* and *prpC*

Ramy K. Aziz[1,2]*, Valerie L. Khaw[2]*, Jonathan M. Monk[2], Elizabeth Brunk[2], Robert Lewis[3], Suh In Loh[4], Arti Mishra[4], Amrita Abhay Nagle[4], Chitkala Satyanarayana[4], Saravanakumar Dhakshinamoorthy[4], Michele Luche[3], Douglas B. Kitchen[3], Kathleen A. Andrews[2], Bernhard Ø. Palsson[2], Pep Charusanti[2,5,§]

[1] Department of Microbiology and Immunology, Faculty of Pharmacy, Cairo University, Cairo, 11562, Egypt; [2] Department of Bioengineering, University of California, San Diego, La Jolla, California, 92093, USA; [3] Computer-Aided Drug Discovery, Albany Molecular Research, Inc., Albany, New York, 12203, USA; [4] Albany Molecular Research Singapore Research Centre, Pte Ltd, Singapore, 117525; [5] The Novo Nordisk Foundation Center for Biosustainability, Technical University of Denmark, Hørsholm, Denmark

* Equal contribution

[§] Corresponding author

CONTENTS

- **Supplementary Figure 1.** Growth rates of each strain in LB and glucose M9 medium.
- **Supplementary Figure 2.** Gel image from screening six additional colonies of the Δ*prpC* Δ*aldA* mutant for the continued presence of *aldA.*
- **Supplementary Figure 3.** PDB files for each site and chemical structures.
- **Supplementary Figure 4.** Hypothesized mechanism to explain synthetic lethality between *aldA* and *prpC.*
- **Supplementary Table 1.** List of 15 genes identified via Tn-mutagenesis as putative synthetic lethal pairs with *aldA*.
- **Supplementary Table 2.** Percent growth inhibition in initial two-by-two combination studies against *E. coli* and *S.* Typhimurium.
- **Supplementary Table 3.** Chemical suppliers and catalog numbers for screening compounds.

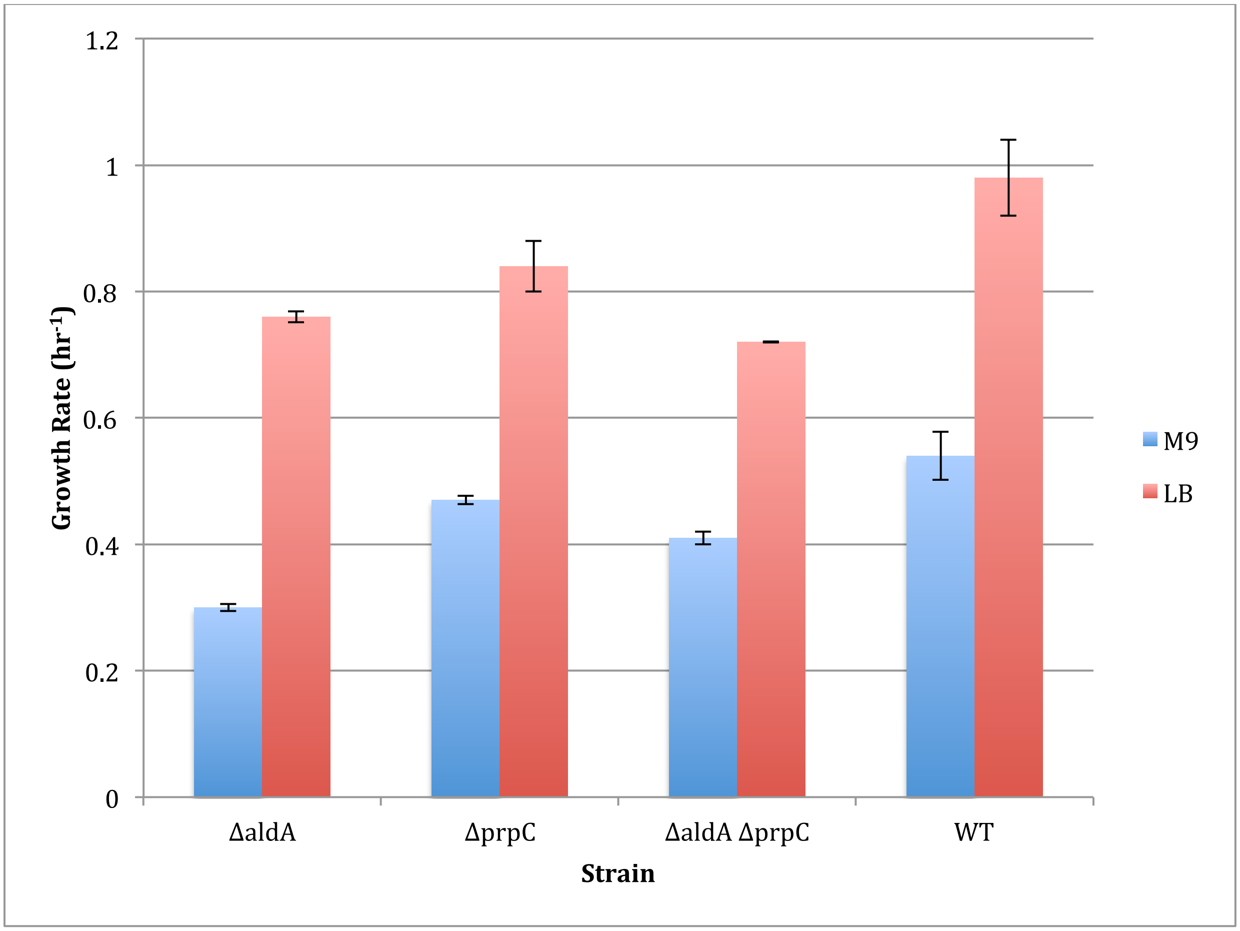

Supplementary Figure 1. Growth rates of each strain in LB and glucose M9 medium. The ΔaldA ΔprpC strain contains a plasmid-borne copy of *aldA*.

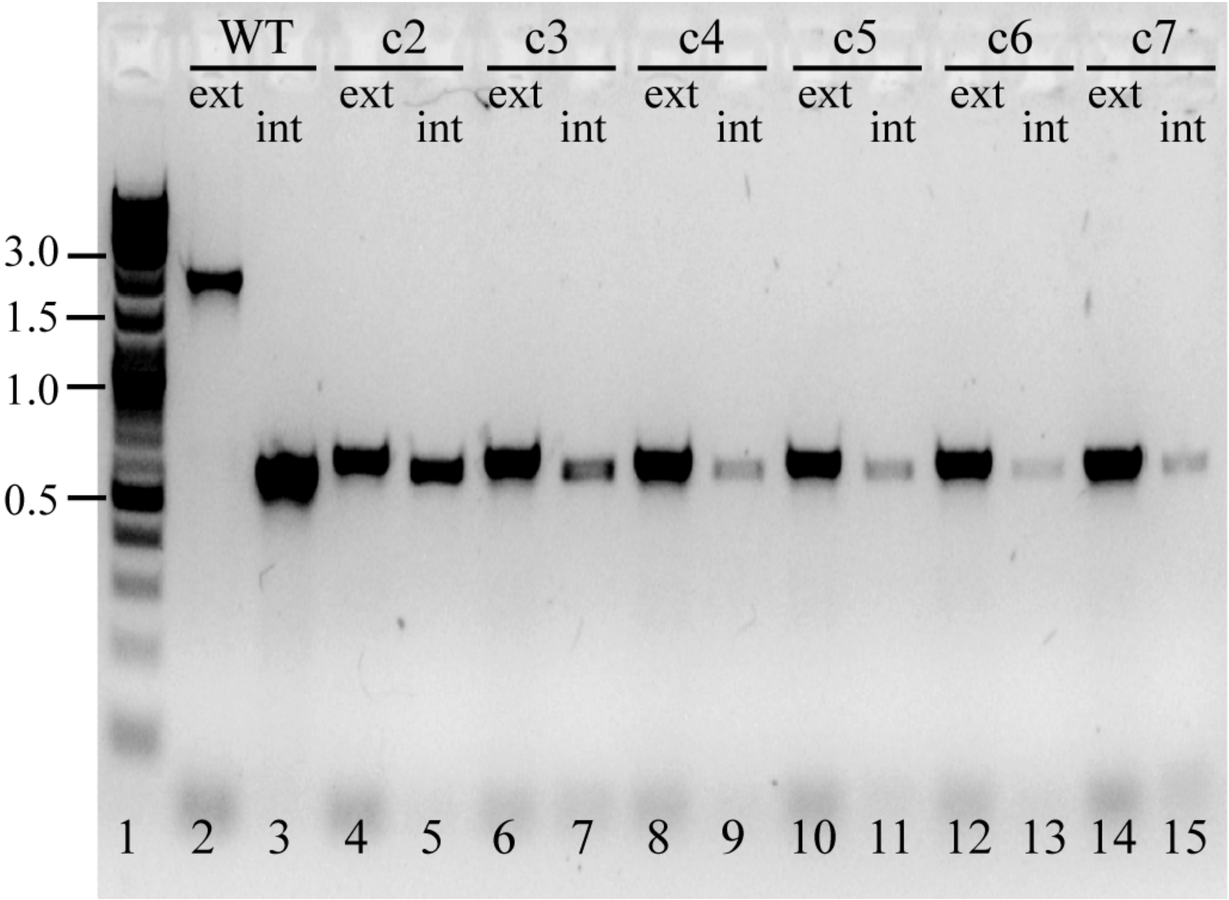

Supplementary Figure 2. Gel image from screening six additional colonies of the Δ*prpC* Δ*aldA* mutant for the continued presence of *aldA*. In addition to the colony seen in Figure 2 of the main text, six additional single colonies of the Δ*prpC* Δ*aldA* double mutant (c2 through c7) were screened to verify deletion of the chromosomal copy of *aldA* using primers that bound upstream and downstream of its annotated position ("ext," or external). All six colonies had the deletion (lanes 4-14, even numbers, versus lane 2). At the same time, PCR amplification using primers that bound wholly within *aldA* also yielded a PCR product, and Sanger sequencing confirmed that it was *aldA* (lanes 5-15, odd numbers). Consequently, each of these mutants continues to possess a copy of *aldA*, but at a different site in its genome.

PRIMERS:

To construct the *aldA* chromosomal deletion:

FWD:

5'-GATGTTAATTAACAATGTATTCACCGAAAACAAACATATAAATCACAGGAGTCGCC
CATGattccggggatccgtcgacc-3'

REV:

5'-ATTCGCTAAACTCTGACGCGCACAGGCGGAGGAAAAAACCTCCGCCTCTTTCAC

TCATTAgtgtaggctggagctgcttc-3'

Confirmation primers:

aldA ext FWD: 5' – GCATGGCAAACGCTGAAACA – 3'

aldA ext REV: 5' – GACTGCCGAAGAGGTGAATAA – 3'

prpC ext FWD: 5' – GGTCGGATAAGACGCATAGC – 3'

prpC ext REV: 5' – TTGAGCTGACATTGGGTACG – 3'

Internal primers for *aldA*:

aldA int FWD: 5' – GGAACTTCCCGTTCTTCCTC – 3'

aldA int REV: 5' – CACTCTCGCCCCTTCTTCTA – 3'

Cloning primers to insert *aldA* into pASK1988:

pASK1988 CPEC FWD: 5' – TAAGCTTGACCTGTGAAGTGA – 3'

pASK1988 CPEC REV: 5' – TTGTATATCTCCTTCTTAAAGT – 3'

aldA CPEC FWD:

5' – TTTGTTTAACTTTAAGAAGGAGATATACAA_ATGTCAGTACCCGTTCAACA – 3'

aldA CPEC REV:

5' – TCACCTCACAGGTCAAGC_TTATTAAGACTGTAAATAAACCACCTG – 3'

Supplementary Figure 3. PDB files for each site

ALDA:

PDB code 2opx was used to develop models with DXC for the ligand.

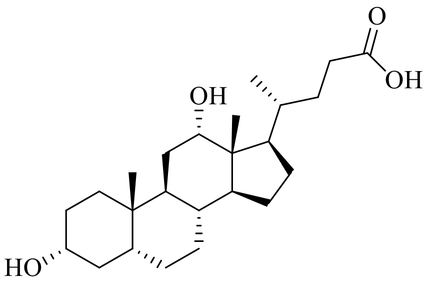

DXC

174 compounds were purchased. Three compounds had measurable IC_{50} values:

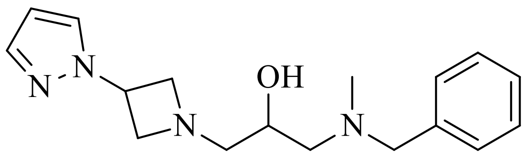

ALDA-112 (112 µM against *E. coli*)

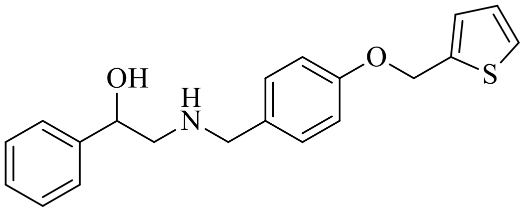

ALDA-170 (187 µM against *E. coli*)

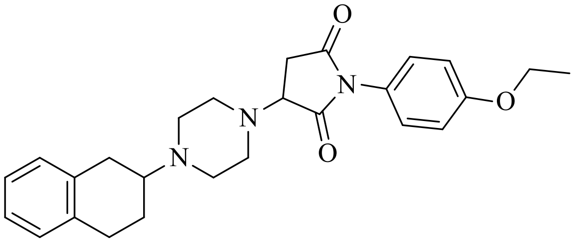

ALDA-087 (24% inhibition at 200 µM against *E. coli*)

PRPC:

PDB code 3o8j was used to develop models with CIC from 6cts as the ligand. The ligand was trimmed down to only the adenosine phosphate for the creation of the model.

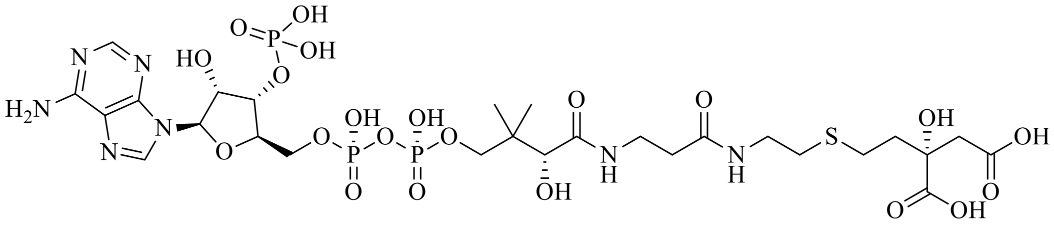

CIC

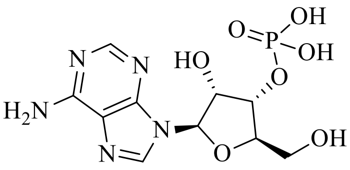

trimmed CIC

100 compounds were purchased. None had measurable IC_{50} values, but one compound did have an observed percent inhibition over 20%:

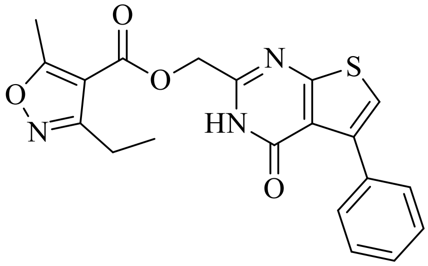

PRPC-034 (27% inhibition at 200 μM against *S.* Typhimurium)

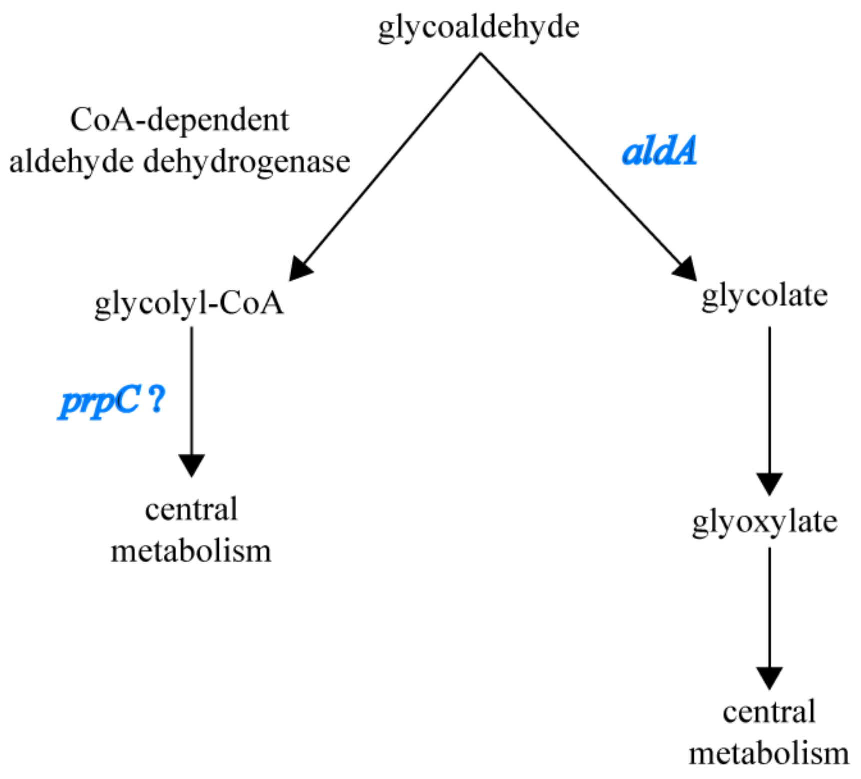

Supplementary Figure 4. Hypothesized mechanism to explain synthetic lethality between *aldA* and *prpC*. AldA is known to convert glycolaldehyde into glycolate (right branch). In model simulations, this reaction is essential because it is the only one that utilizes glycolaldehyde. Without this reaction, an infinite amount of glycolaldehyde would accumulate, which is an infeasible solution. An alternate utilization pathway is hypothesized here to exist that involves *prpC* (left branch). In this hypothetical pathway, glycolaldehyde is first converted into glycolyl-CoA by an unknown coenzyme A-dependent aldehyde dehydrogenase. Next, PrpC would convert glycolyl-CoA into metabolites that could enter central metabolism. The two genes *aldA* and *prpC* are synthetically lethal as their removal shuts down both pathways that utilize glycolaldehyde. The unknown coenzyme A-dependent aldehyde dehydrogenase should also be synthetically lethal with *aldA,* but no such enzyme was discovered during the Tn-mutagenesis screen. This outcome suggests that Tn-mutagenesis did not saturate the genome sufficiently to detect all genes, or that more than one enzyme can catalyze this step.

Supplementary Table 1. List of 15 genes identified by Tn-mutagenesis as putative synthetic lethal pairs with *aldA*. Only *aldA* / *prpC* was confirmed as a synthetic lethal pair. For all others, defined double knockout mutants could be created and grown in both LB and glucose M9*.*

Gene	Locus tag	Function
prpC	b0333	2-methylcitrate synthase
yhfs	b3376	FNR-regulated pyridoxal phosphate-dependent
yfaw / *rhmD*	b2247	L-rhamnonate dehydratase
ypeA	b2434	GNAT family putative N-acetyltransferase
speA	b2938	arginine decarboxylase
hypE	b2730	carbamoyl dehydratase, hydrogenases 1,2,3 maturation protein
ydjK	b1775	putative transporter
ygeX	b2871	2,3-diaminopropionate ammonia-lyase
pabC	b1096	aminodeoxychorismate lyase; component of pABA synthase
pqiA	b0950	paraquat-inducible, SoxRS-regulated inner membrane protein
cadA	b4131	lysine decarboxylase
rspA	b1581	bifunctional D-altronate / D-mannonate dehydratase
casA	b2760	CRISPR-associated gene
ycaM	b0899	putative transporter
ybiX	b0804	putative Fe(II)-dependent oxygenase superfamily protein

Supplementary Table 2. Percent growth inhibition in initial two-by-two combination studies against (A) *E. coli* and (B) *S.* Typhimurium. The actual percent growth inhibitions achieved by testing two compounds in combination were compared with their expected additive values according to the Loewe model. The data are shown along with the calculated standard deviation. Values shown in bold indicate potential synergistic concentrations. The concentrations of each compound used in the two-by-two tests are shown in (C). The asterisk denotes compounds for which IC_{50} values could not be determined.

(A)

	***E. coli* Preliminary Combination Study**							
	**Actual Percent Growth Inhibition**				**Expected Percent Growth Inhibition (if additive effect)**			
**Combination A + B**	A1+B1	A1+B2	A2+B1	A2+B2	A1+B1	A1+B2	A2+B1	A2+B2
ALDA-087 + ALDA-112	18.73 ± 0.24	6.71 ± 1.1	21.67 ± 0.05	8.23 ± 1.77	23.3	7.5	25.7	9.8
ALDA-087 + ALDA-170	47.05 ± 2.05	19.76 ± 1.45	50.09 ± 0.23	20.74 ± 0.54	53.4	19	55.8	21.4
ALDA-087 + PRPC-034	11.78 ± 0.21	5.90 ± 0.75	7.10 ± 2.24	5.93 ± 1.17	11.5	11.1	13.8	13.5
ALDA-112 + ALDA-170	**99.73 ± 0.09**	38.88 ± 1.29	56.86 ± 1.99	23.78 ± 2.43	**82.8**	48.4	66.9	32.5
ALDA-112 + PRPC-034	29.33 ± 4.14	29.49 ± 4.85	19.35 ± 5.08	20.09 ± 5.89	40.9	40.5	25	24.6
ALDA-170 + PRPC-034	49.94 ± 2.21	51.95 ± 2.52	33.35 ± 1.51	33.86 ± 4.28	70.9	70.6	36.5	36.2

(B)

	***S.* Typhimurium Preliminary Combination Study**							
	**Actual Percent Growth Inhibition**				**Expected Percent Growth Inhibition (if additive effect)**			
**Combination A + B**	A1+B1	A1+B2	A2+B1	A2+B2	A1+B1	A1+B2	A2+B1	A2+B2
ALDA-087 + ALDA-112	-13.53 (+/-) 3.1	-6.58 (+/-) 7.46	-10.02 (+/-) 13.54	-12.85 (+/-) 26.86	21.2	15.4	24	18.2
ALDA-087 + ALDA-170	29.38 ± 9.06	17.53 ± 0.70	40.45 ± 0.09	15.54 ± 11.01	49.8	38	52.6	40.7
ALDA-087 + PRPC-034	14.37 ± 6.99	19.89 ± 7.5	20.42 ± 3.5	11.36 ± 17.52	27.9	42.9	30.6	45.7
ALDA-112 + ALDA-170	**42.69 ± 0.16**	9.59 ± 13.74	25.09 ± 7.7	-7.28 (+/-) 5.94	**40**	28.1	34.2	22.3
ALDA-112 + PRPC-034	**24.82 ± 5.17**	31.14 ± 2.14	18.68 ± 1.14	22.37 ± 2.97	**18.1**	33.1	12.3	27.3
ALDA-170 + PRPC-034	7.60 ± 8.9	17.35 ± 13.43	-7.66 (+/-) 9.39	-24.37 (+/-) 11.14	46.7	61.7	34.8	49.9

(C)

	Selected Concentration (µM)	
**Compound ID**	**1**	**2**
ALDA-087*	200	100
ALDA-112	100	50
ALDA-170	200	100
PRPC-034*	200	100

Supplementary Table 3. Chemical suppliers and catalog numbers for screening compounds.

Compound ID	Vendor	Catalog No.
ALDA-001	ChemBridge	51483769
ALDA-002	ChemBridge	18030242
ALDA-003	ChemBridge	32375885
ALDA-004	ChemBridge	50970311
ALDA-005	ChemBridge	56323167
ALDA-006	ChemBridge	43142476
ALDA-007	ChemBridge	89218124
ALDA-008	ChemBridge	66974801
ALDA-009	ChemBridge	76990033
ALDA-010	ChemBridge	64296819
ALDA-011	ChemBridge	40740714
ALDA-012	ChemBridge	65155043
ALDA-013	ChemBridge	31344667
ALDA-014	ChemBridge	61284609
ALDA-015	ChemBridge	17624247
ALDA-016	ChemBridge	95751814
ALDA-017	ChemBridge	29862172
ALDA-018	ChemBridge	39887131
ALDA-019	ChemBridge	63087467
ALDA-020	ChemBridge	18741551
ALDA-021	ChemBridge	47315314
ALDA-022	ChemBridge	45091455
ALDA-023	ChemBridge	25029473
ALDA-024	ChemBridge	71838273
ALDA-025	ChemBridge	26386310
ALDA-026	ChemBridge	22359149
ALDA-027	ChemBridge	86524019
ALDA-028	ChemBridge	21902084
ALDA-029	ChemBridge	56896973
ALDA-030	ChemBridge	79000372
ALDA-031	ChemBridge	14214075
ALDA-032	ChemBridge	31406938
ALDA-033	ChemBridge	46252436
ALDA-034	ChemBridge	97685253
ALDA-035	ChemBridge	38051710

Compound ID	Vendor	Catalog No.
ALDA-036	ChemBridge	56000302
ALDA-037	ChemBridge	37686732
ALDA-038	ChemBridge	25539447
ALDA-039	ChemBridge	34871648
ALDA-040	ChemBridge	20862459
ALDA-041	ChemBridge	93468938
ALDA-042	ChemBridge	30900904
ALDA-043	ChemBridge	45102073
ALDA-044	ChemBridge	48706196
ALDA-045	ChemBridge	15025066
ALDA-046	ChemBridge	84343165
ALDA-047	ChemBridge	51193726
ALDA-048	ChemBridge	13780944
ALDA-049	ChemBridge	83044248
ALDA-050	ChemBridge	35835700
ALDA-051	ChemBridge	13315330
ALDA-052	ChemBridge	30087128
ALDA-053	ChemBridge	47177753
ALDA-054	ChemBridge	25030516
ALDA-055	ChemBridge	65533887
ALDA-056	ChemBridge	44868717
ALDA-057	ChemBridge	28198596
ALDA-058	ChemBridge	55830528
ALDA-059	ChemBridge	27696084
ALDA-060	ChemBridge	19452112
ALDA-061	ChemBridge	22811531
ALDA-062	ChemBridge	84604140
ALDA-063	ChemBridge	55098641
ALDA-064	ChemBridge	54447001
ALDA-065	ChemBridge	54415264
ALDA-066	ChemBridge	10304738
ALDA-067	ChemBridge	70432875
ALDA-068	ChemBridge	98982380
ALDA-069	ChemBridge	24271115
ALDA-070	ChemBridge	82040370
ALDA-071	ChemBridge	35136054
ALDA-072	ChemBridge	90718785
ALDA-073	ChemBridge	18745783

Compound ID	Vendor	Catalog No.
ALDA-074	ChemBridge	49709985
ALDA-075	ChemBridge	15417220
ALDA-076	ChemBridge	76735799
ALDA-077	ChemBridge	72648632
ALDA-078	ChemBridge	34547760
ALDA-079	ChemBridge	91723715
ALDA-080	ChemBridge	94467574
ALDA-081	ChemBridge	58461145
ALDA-082	ChemBridge	9107029
ALDA-083	ChemBridge	9102507
ALDA-084	ChemBridge	9039270
ALDA-085	ChemBridge	7958751
ALDA-086	ChemBridge	7909489
ALDA-087	ChemBridge	7174879
ALDA-088	ChemBridge	6695638
ALDA-089	ChemBridge	5913895
ALDA-090	ChemBridge	5546245
ALDA-091	ChemBridge	5307654
ALDA-092	ChemBridge	5254845
ALDA-093	Enamine	Z968660346
ALDA-094	Enamine	Z1139742472
ALDA-095	Enamine	Z52205166
ALDA-096	Enamine	Z318884182
ALDA-097	Enamine	Z26716554
ALDA-098	Enamine	Z432765662
ALDA-099	Enamine	Z643021442
ALDA-100	Enamine	Z1139284893
ALDA-101	Enamine	Z1139574848
ALDA-102	Enamine	Z1139273670
ALDA-103	Enamine	Z217581414
ALDA-104	Enamine	Z374870146
ALDA-105	Enamine	Z641505038
ALDA-106	Enamine	Z1139742128
ALDA-107	Enamine	Z1024744838
ALDA-108	Enamine	Z229384966
ALDA-109	Enamine	Z126472220
ALDA-110	Enamine	Z46185444
ALDA-111	Enamine	Z374612766

Compound ID	Vendor	Catalog No.
ALDA-112	Enamine	Z1139549184
ALDA-113	Enamine	Z1139507212
ALDA-114	Enamine	Z109705808
ALDA-115	Enamine	Z667598336
ALDA-116	Enamine	Z1007582548
ALDA-117	Enamine	Z646608158
ALDA-118	Enamine	Z1139743732
ALDA-119	Enamine	Z1393302898
ALDA-120	Enamine	Z1497518442
ALDA-121	Enamine	Z1592757987
ALDA-122	Enamine	Z1139260026
ALDA-123	Enamine	Z1074714796
ALDA-124	Enamine	Z1171367576
ALDA-125	Enamine	Z967951916
ALDA-126	Enamine	Z1139363421
ALDA-127	Enamine	Z31383295
ALDA-128	Enamine	Z996483908
ALDA-129	Enamine	Z1119755156
ALDA-130	Enamine	Z1139273740
ALDA-131	Enamine	Z1139404818
ALDA-132	Enamine	Z643988250
ALDA-133	Enamine	Z1139276090
ALDA-134	Enamine	Z1002967880
ALDA-135	Enamine	Z1002968496
ALDA-136	Enamine	Z267954988
ALDA-137	Enamine	Z1002968580
ALDA-138	Enamine	Z1139274645
ALDA-139	Enamine	Z367566102
ALDA-140	Enamine	Z1147459342
ALDA-141	Enamine	Z989734454
ALDA-142	Enamine	Z608816070
ALDA-143	Enamine	Z109710514
ALDA-144	Enamine	Z385254612
ALDA-145	Enamine	Z384484862
ALDA-146	Enamine	Z59740361
ALDA-147	Enamine	Z223841982
ALDA-148	Enamine	Z46480145
ALDA-149	Enamine	Z167651438

Compound ID	Vendor	Catalog No.
ALDA-150	Enamine	Z252287358
ALDA-151	Enamine	Z105649074
ALDA-152	Enamine	Z28582729
ALDA-153	Enamine	Z188951980
ALDA-154	Enamine	Z62828371
ALDA-155	Enamine	Z31367519
ALDA-156	Enamine	Z105650634
ALDA-157	Enamine	Z44097741
ALDA-158	Enamine	Z27018642
ALDA-159	Enamine	Z31968506
ALDA-160	Enamine	Z130300706
ALDA-161	Princeton BioMolecular Research	OSSL_428490
ALDA-162	Princeton BioMolecular Research	OSSL_048037
ALDA-163	Princeton BioMolecular Research	OSSK_978300
ALDA-164	Princeton BioMolecular Research	OSSK_674224
ALDA-165	Princeton BioMolecular Research	OSSK_601786
ALDA-166	Princeton BioMolecular Research	OSSK_514007
ALDA-167	Princeton BioMolecular Research	OSSL_648517
ALDA-168	Princeton BioMolecular Research	OSSL_049087
ALDA-169	Princeton BioMolecular Research	OSSK_685957
ALDA-170	Princeton BioMolecular Research	OSSK_999213
ALDA-171	Princeton BioMolecular Research	OSSL_391814
ALDA-172	Princeton BioMolecular Research	OSSK_938369
ALDA-173	Princeton BioMolecular Research	OSSK_817102
ALDA-174	Princeton BioMolecular Research	OSSK_817100
PRPC-001	ChemBridge	32226558
PRPC-002	ChemBridge	12319495
PRPC-003	ChemBridge	25873940
PRPC-004	ChemBridge	31130923
PRPC-005	ChemBridge	22571303
PRPC-006	ChemBridge	11336339
PRPC-007	ChemBridge	9298668
PRPC-008	ChemBridge	9129687
PRPC-009	ChemBridge	9112797
PRPC-010	ChemBridge	9037539
PRPC-011	ChemBridge	9026046
PRPC-012	ChemBridge	7990881
PRPC-013	ChemBridge	7164939

Compound ID	Vendor	Catalog No.
PRPC-014	ChemBridge	5754262
PRPC-015	Enamine	Z56834695
PRPC-016	Enamine	Z1185959286
PRPC-017	Enamine	Z228823892
PRPC-018	Enamine	Z126933868
PRPC-019	Enamine	Z666183288
PRPC-020	Enamine	Z357940036
PRPC-021	Enamine	Z1262693378
PRPC-022	Enamine	Z26414390
PRPC-023	Enamine	Z1444490356
PRPC-024	Enamine	Z27690782
PRPC-025	Enamine	Z134945162
PRPC-026	Enamine	Z90268017
PRPC-027	Enamine	Z30168412
PRPC-028	Enamine	Z238637042
PRPC-029	Enamine	Z29941821
PRPC-030	Enamine	Z18545792
PRPC-031	Enamine	Z29941978
PRPC-032	Enamine	Z54365716
PRPC-033	Enamine	Z17141521
PRPC-034	Enamine	Z74644270
PRPC-035	Enamine	Z89126247
PRPC-036	Enamine	Z29939820
PRPC-037	Enamine	Z57015354
PRPC-038	Enamine	Z14277235
PRPC-039	Enamine	Z666292414
PRPC-040	Enamine	Z113828246
PRPC-041	Enamine	Z651070284
PRPC-042	Enamine	Z85891019
PRPC-043	Enamine	Z169681404
PRPC-044	Enamine	Z1142783502
PRPC-045	Enamine	Z1139523845
PRPC-046	Enamine	Z1143007577
PRPC-047	Enamine	Z1168473822
PRPC-048	Enamine	Z822250674
PRPC-049	Enamine	Z942817544
PRPC-050	Enamine	Z954493758
PRPC-051	Enamine	Z485320854

Compound ID	Vendor	Catalog No.
PRPC-052	Enamine	Z92682804
PRPC-053	Enamine	Z100584040
PRPC-054	Enamine	Z359440278
PRPC-055	Enamine	Z27794039
PRPC-056	Enamine	Z29942141
PRPC-057	Enamine	Z30848450
PRPC-058	Enamine	Z374055464
PRPC-059	Enamine	Z100584036
PRPC-060	Enamine	Z839042800
PRPC-061	Enamine	Z649731960
PRPC-062	Enamine	Z24640843
PRPC-063	Enamine	Z403712634
PRPC-064	Enamine	Z371619698
PRPC-065	Enamine	Z29694163
PRPC-066	Enamine	Z70910314
PRPC-067	Enamine	Z279857278
PRPC-068	Enamine	Z19025546
PRPC-069	Enamine	Z80297397
PRPC-070	Enamine	Z29967618
PRPC-071	Enamine	Z14279450
PRPC-072	Enamine	Z29245339
PRPC-073	Enamine	Z57015377
PRPC-074	Enamine	Z90292619
PRPC-075	Enamine	Z109712132
PRPC-076	Princeton BioMolecular Research	OSSL_451918
PRPC-077	Princeton BioMolecular Research	OSSL_121303
PRPC-078	Princeton BioMolecular Research	OSSL_051177
PRPC-079	Princeton BioMolecular Research	OSSK_777530
PRPC-080	Princeton BioMolecular Research	OSSK_777285
PRPC-081	Princeton BioMolecular Research	OSSK_570392
PRPC-082	Princeton BioMolecular Research	OSSK_529320
PRPC-083	Princeton BioMolecular Research	OSSK_327437
PRPC-084	Princeton BioMolecular Research	OSSL_648988
PRPC-085	Princeton BioMolecular Research	OSSL_623810
PRPC-086	Princeton BioMolecular Research	OSSL_622118
PRPC-087	Princeton BioMolecular Research	OSSL_616765
PRPC-088	Princeton BioMolecular Research	OSSL_601297
PRPC-089	Princeton BioMolecular Research	OSSL_601251

Compound ID	Vendor	Catalog No.
PRPC-090	Princeton BioMolecular Research	OSSL_158054
PRPC-091	Princeton BioMolecular Research	OSSK_531230
PRPC-092	Princeton BioMolecular Research	OSSK_531176
PRPC-093	Princeton BioMolecular Research	OSSK_090103
PRPC-094	Princeton BioMolecular Research	OSSL_123093
PRPC-095	Princeton BioMolecular Research	OSSK_790129
PRPC-096	Princeton BioMolecular Research	OSSL_369619
PRPC-097	Princeton BioMolecular Research	OSSK_773948
PRPC-098	Princeton BioMolecular Research	OSSK_488906
PRPC-099	Princeton BioMolecular Research	OSSK_897146
